# Leader manipulator with hand rest function for microsurgery

**DOI:** 10.1002/rcs.2355

**Published:** 2021-12-08

**Authors:** Solmon Jeong, Kotaro Tadano

**Affiliations:** ^1^ School of Engineering Tokyo Institute of Technology Yokohama‐shi Japan; ^2^ Laboratory for Future Interdisciplinary Research of Science and Technology Tokyo Institute of Technology Yokohama‐shi Japan

**Keywords:** hand rest, leader manipulator, microsurgery, robotic surgery, teleoperation

## Abstract

**Background:**

Hand rest assists microsurgeons with maintaining a stable hand condition. In manipulating a surgical robot using teleoperation, the external hand rest poses mechanical difficulties such as interferences from manipulators.

**Methods:**

Herein, we introduced hand rest functionality into a leader manipulator. The hand rest function was realised by switching the impedance value in admittance control for the translation section. With teleoperation, we evaluated the positioning accuracy in the direction of gravity and the manipulating conditions during precise positioning.

**Results:**

The experiments proved that the position of the hand rest could be arranged at the intended position, and the condition with the hand rest function, applying sufficiently high impedance, and not constraining the hand rest completely provided the best performance.

**Conclusion:**

Our leader manipulator showed the potentials to improve the quality of robotic microsurgery with the advantage in fine manipulation based on large repositionable working range of the hand rest.

## 1 INTRODUCTION

Microsurgery is a form of surgery that provides microscale precision in otolaryngology, brain surgeries and other surgeries. Microsurgical procedures performing submillimetric tasks are extremely difficult to perform, even for skilled surgeons, and the success rate depends mostly on the surgeon's level of experience.^1^ With the magnified operating field through a microscope, surgeons perform microsurgical procedures by moving their wrists and fingers while stabilising their hands. In other words, precise wrist and finger movements based on stable hand positions are important elements of microsurgical procedures.^2^

One main challenge for the future of microsurgery is to perform tasks beyond human perception and abilities.^3^ With the advantages of a surgical robot system using teleoperation, that is, leader–follower operation such as a scalable motion control and hand tremor filtering, surgeons can improve the dexterity and precision.^4^ Since the introduction of the surgical robot, several microsurgical developments have been demonstrated through improved robot‐assisted surgical technologies, such as the da Vinci system, RobOtol and MicroSure's MUSA.^5^, ^6^, ^7^ Despite the potential of a surgical robot to overcome the limitations of conventional surgery, robotic surgeons can experience physical symptoms or discomfort, such as finger fatigue, and robot‐assisted procedures take longer than manual operation.^8^ ^,^ ^9^ The prolonged operation duration increases the risk of complications, such as flap failure, wound dehiscence and infection.^10^ Motion scaling with a larger scale factor in teleoperation requires bigger input motion for precise operation, causing further time extension. Hence, operating the surgical robot with the smallest scale factor possible is also desired.^11^ Moreover, the robotic surgeon tends to manipulate the leader manipulator slower than expected for safe performance in teleoperation, whereas the robot‐assisted and manual procedures share almost the same surgical scenarios in surgical tool motion.^12^

People often require ergonomic support for their hands to increase precision while performing dexterous tasks, and hand rest is indispensable in microsurgery for reducing hand tremor.^13^ Similarly, the use of hand rest helps to improve precision and reduce muscle fatigue in manipulating the leader manipulator of robotic surgery.^14^ Despite these advantages, static hand rest can be used in only small workspaces and repositioning the hand rest requires additional efforts for optimisation. Particularly, for a microsurgical procedure, these efforts may interfere with the entire procedure. An easily repositionable hand rest improves the precision and speed of surgical tasks.^15^ Moreover, repositionable hand rest can create multiple small dexterous workspaces. iArmS has proved its effectiveness in terms of accuracy and safety in clinical applications.^16^ Similarly, the active hand rest for a haptic device operator has shown promising results in terms of precision and fatigue on operators.^17^ However, equipment operating separately from the leader manipulator may interfere with it or could be difficult to use in a limited operating environment. Thus, integrating the motion input and hand rest functions into a leader manipulator is desired.

In this study, we present a leader manipulator that enables wrist and finger movements under a stable hand position, which is essential in manual microsurgical procedures. Our leader manipulator integrates the motion input and hand rest functions. We performed pointing experiments under several finger and hand movement conditions after evaluating the positioning accuracy of the hand rest function in the direction of gravity. We verified the most effective operating condition of the hand rest function on the basis of the number of failures, time required, trajectory of hand and fingertip, and preference on the manipulating condition.

## 2 MATERIALS AND METHODS

### 2.1 Concept of a leader manipulator with a hand rest

Pinch or power grips have been adopted in manipulating a leader manipulator, based on their advantages for precise or coarse works.^18^ In our previous study, we proposed a combined‐grip scheme of pinch and power grips for mechanically manipulating a leader manipulator. We previously verified the effectiveness of a combined‐grip scheme in positioning operation. In the previously proposed manipulator, the operator inputs the motion by two fingertips on a plane, named the pinch grip motion plane, while holding the handle for the power grip by the entire hand. In this framework, ergonomic access considering the finger movement and introduction of the hand rest, which is adjustable in all directions, were considered as the challenges.^19^

**FIGURE 1:**
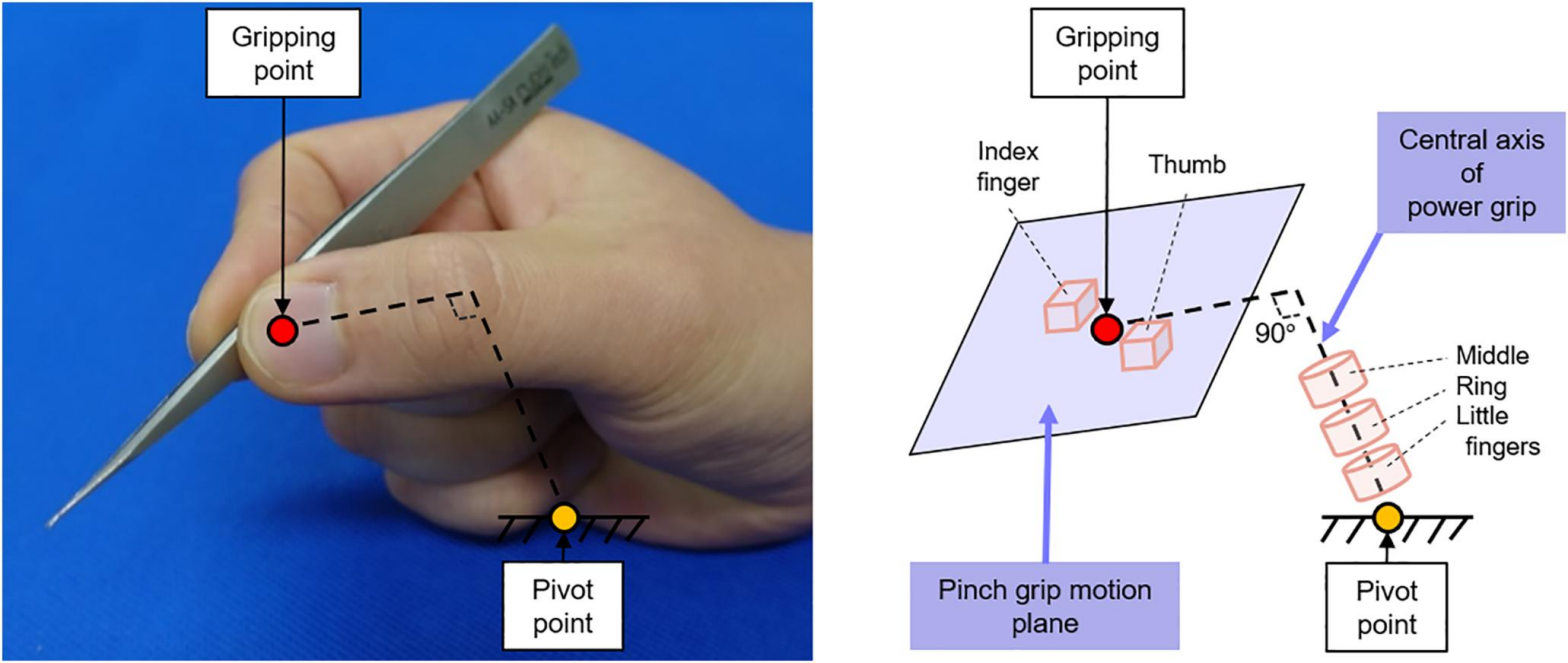
Overview of a hand holding a surgical tool

Hand rest provides ergonomic support during surgery, reduces fatigue and increases precision. Generally, it supports the loads caused by the hand and provides a stable hand position. In the practical microsurgery training, trainees perform the training procedures around the fixed section of the hand, such as the little finger immobilised by rubber bands attached to a support.^20^ Figure 1 shows an example of a hand holding a surgical tool. In this study, we assumed that the movement of the entire hand on the hand rest corresponds to a pivot movement around the contact point between the hand and hand rest. In other words, the position of the pivot point is the position of the hand rest, and determining the position of the pivot point equates to the initial setting of a hand rest in the conventional surgery.

In this study, we propose a leader manipulator, which integrates the motion input and hand rest functions. Considering the surgical procedure with the hand rest, the gripping point of the surgical tool is operated with 7 degrees of freedom (DoFs), after the hand rest is placed with 3 DoFs in translation. Based on the presented background, the main concepts of developing a leader manipulator in this study are as follows:

- To implement the hand rest function to be repositioned in all directions.
- To allow a precise work by fingertips based on the stabilised hand condition.

### 2.2 Design of the leader manipulator

**FIGURE 2:**
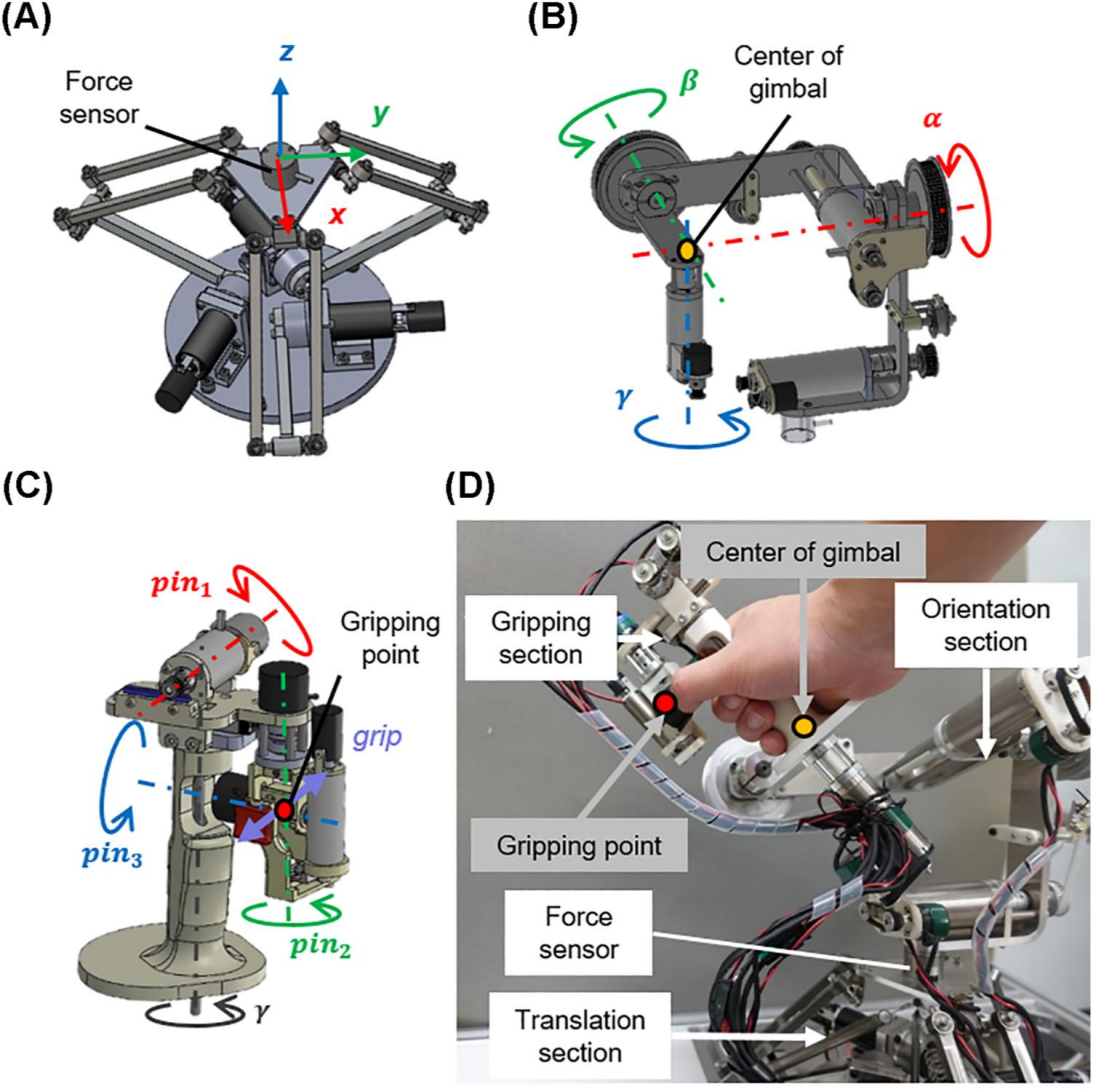
Overview of the leader manipulator consisting of (A) translation, (B) orientation and (C) gripping sections. (D) Photograph of the leader manipulator

The proposed leader manipulator shown in Figure 2 has a total of 10 DoFs, consisting of the translation (3 DoFs), orientation (3 DoFs) and gripping (4 DoFs) sections. We adopted a delta mechanism as the translation section for high positioning accuracy and a force sensor (BL AUTOTEC, LTD, MICRO 4/30‐A) is mounted on the top of the translation section. A serial gimbal mechanism in which three axes intersect at the centre of the gimbal is adopted as the orientation section on the top of the force sensor. The gripping section, which is mounted on the orientation section, consists of 2 DoFs ($pin_{1},\;grip$) with active joints and 2 DoFs ($pin_{2},\;pin_{3}$) with passive joints using only encoders. In the translation section, we used harmonic drive gears (Harmonic Drive Systems, INC, CSF‐2XH Series) as reduction gears, and the backlash of each motor was negligibly small. In the orientation and gripping sections, we used low reduction with the pulley and timing belt or no reduction for high back drivability.

**FIGURE 3:**
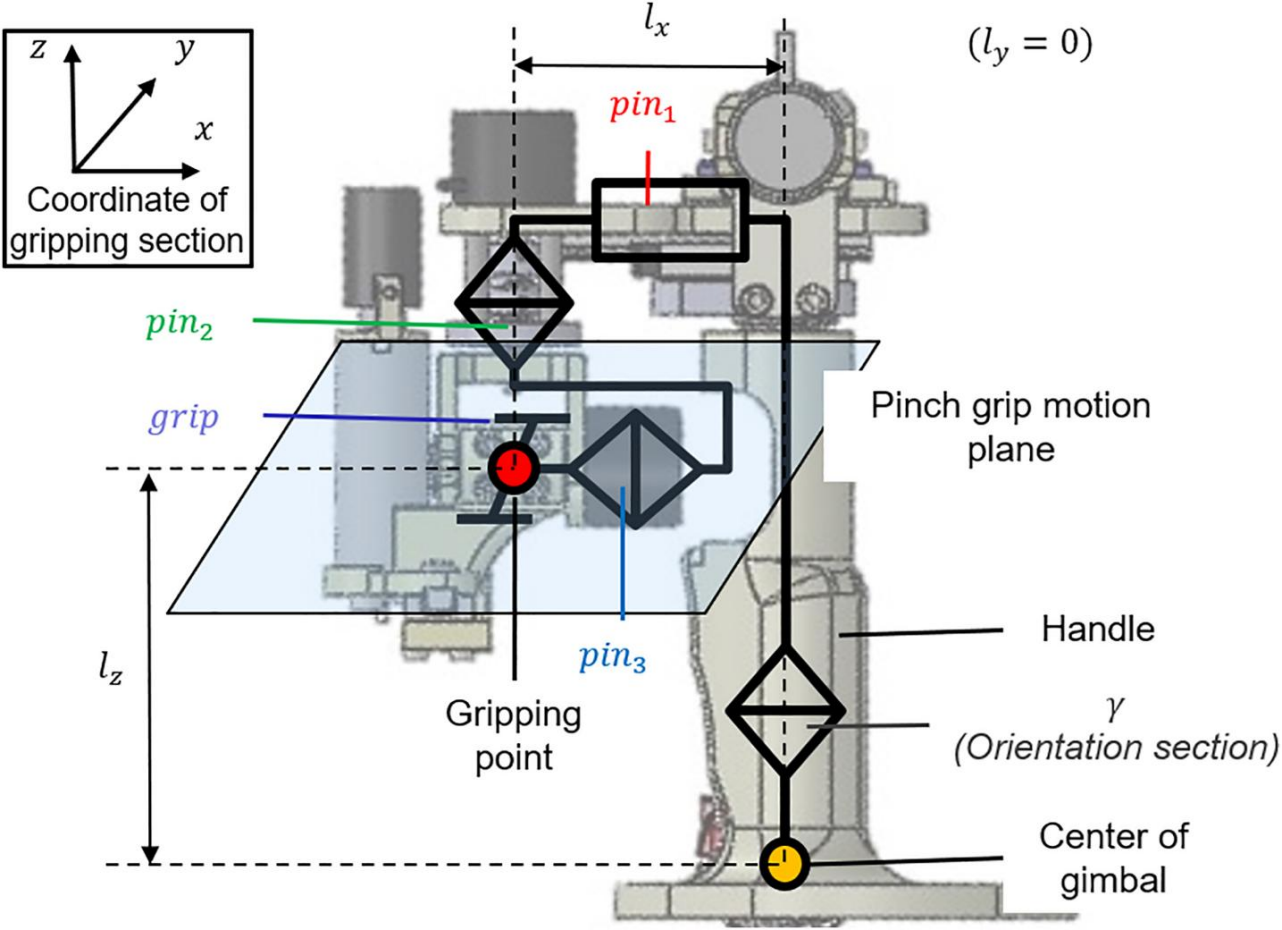
Hardware design of the gripping section

Figure 3 shows the hardware design of the gripping section. In cooperation with the γ‐axis of the orientation section, the position and posture between the gripping point and the central axis (γ) of the handle are adjustable on the pinch grip motion plane. During the operation, the gripping point can be manipulated with the thumb and index fingertips, and the handle is held with the other three fingers. The handle can be passively and separately adjusted using a couple of bearings, regardless of the rotation of the central axis. The axes of $pin_{2}$ and $pin_{3}$ intersect at the gripping point, and the rotations of them are not related to the positional information. Based on the symbols in Figure 3, the positional and postural information of the gripping section and the total input motion in the leader were obtained as follows:

(1) $$X_{g}\;=\;{\left(-l_{x}\;l_{y}\;l_{z}\right)}^{T}$$

(2) $$X_{m}\;=\;R_{o}\;\cdot \;X_{g}\;+\;X_{t}$$

(3) $$R_{g}\;=\;R_{\mathrm{pin}2}\;\cdot \;R_{\mathrm{pin}3}$$

(4) $$R_{m}\;=\;R_{o}\;\cdot \;R_{g}$$

where $X_{g}$ is the position of the gripping point in the coordinate of the gripping section; $X_{t}$ and $X_{m}$ are the positions of the translation section and total input in the leader at Cartesian coordinates; $R_{\mathrm{pin}2}$, $R_{\mathrm{pin}3}$, and $R_{g}$ are the rotation matrices from the axes of $pin_{2}$, $pin_{3}$ and gripping section; and $R_{o}$ and $R_{m}$ are the rotation matrices from the orientation section and of the total input in the leader.

### 2.3 Control system and hand rest function of the leader manipulator

The orientation and gripping sections are separated from the translation section in control as well as in hardware. The centre of the gimbal in the orientation section, which works as a pivot point, depends on only the movement of the translation section. We adopted impedance control for the orientation and gripping sections with high back drivability and admittance control for the translation section using the external force measured by the force sensor. Using the admittance control, the translation section was controlled and operated through virtual model dynamics to achieve a desirable interaction with an operator by measuring the interaction force.^21^ The force–velocity relation for controlling the translation section followed the equations below:

(5) $${\dot{X}}_{t}\left(s\right)\;=\;\frac{1}{Z\left(s\right)}\cdot F\left(s\right)$$

(6) Z(s)=M⋅s+B

where ${\dot{X}}_{t}\left(s\right)$ is the reference velocity, F(s) is the external force, Z(s) is the impedance (virtual dynamics), and M and B represent the inertia and viscosity constructing the impedance, respectively.

**FIGURE 4:**
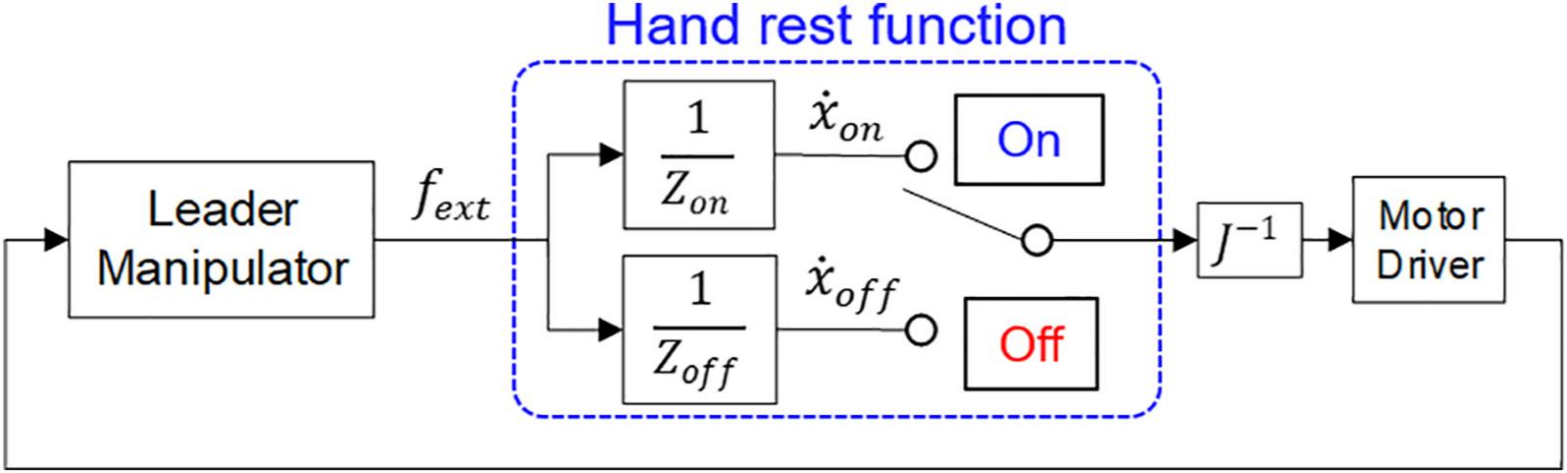
Control system of the translation section for hand rest function: $f_{\text{ext}}$, external force; J, Jacobian matrix of the translation section; $Z_{\text{on}},\;Z_{\text{off}}$, impedance value in each mode; ${\dot{x}}_{\text{on}},{\dot{x}}_{\text{off}}$, reference velocity in each mode

**FIGURE 5:**
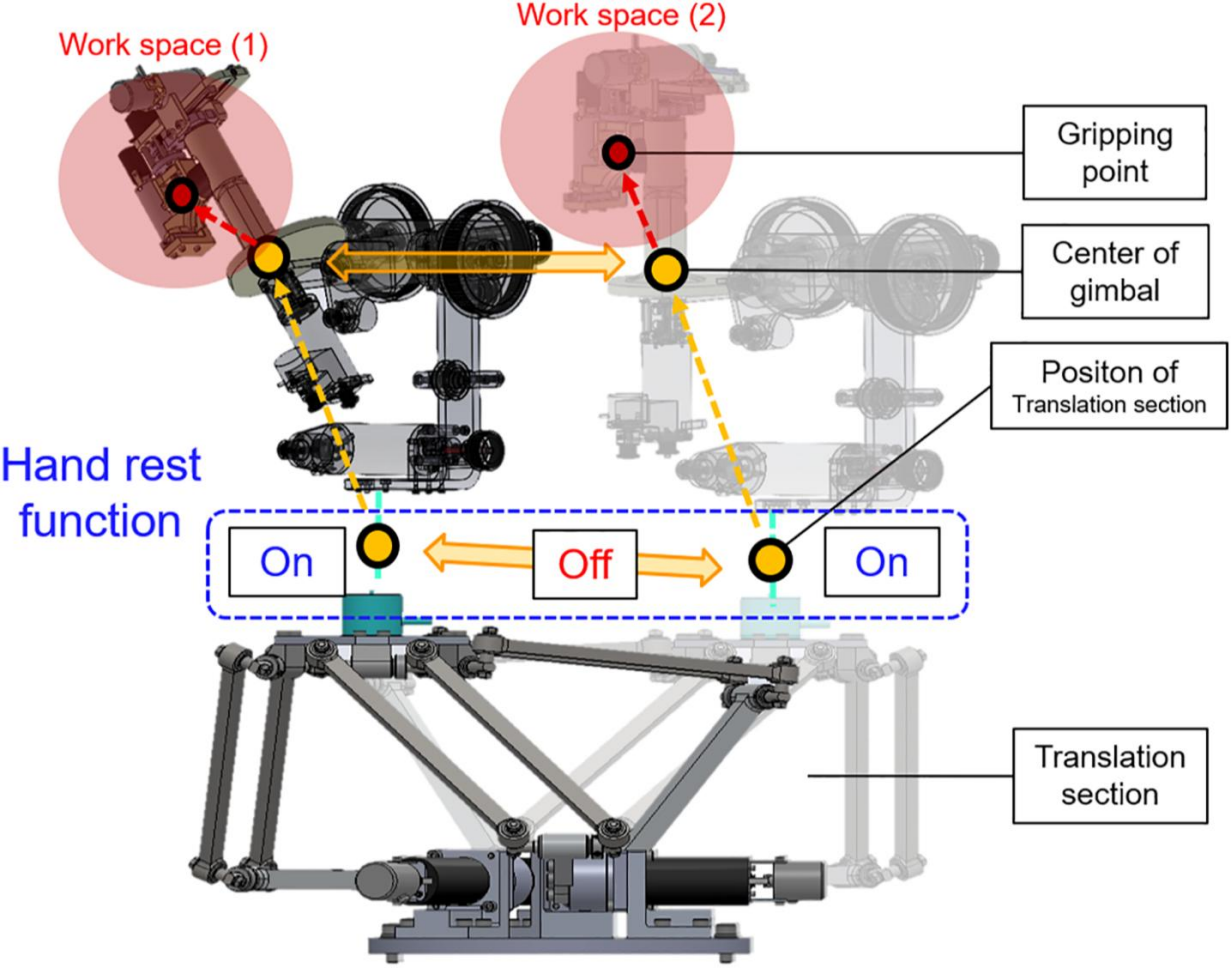
Overview of operating the hand rest function

In this study, we realised the hand rest function by switching virtual dynamics, that is, the impedance in admittance control. When a considerably high impedance value is applied, the reference velocity approaches zero; thus, the translation section stays in the position although an external force is applied. As shown in Figure 4, the hand rest function operates in on and off modes. In the off mode, an operator can transport the pivot point freely by manipulating the translation section with desired impedance ($Z_{\text{off}}$). In the on mode, a considerably high impedance ($Z_{\text{on}}$) is applied, and the translation section stays in that position, providing the operator with the stable pivot point. As shown in Figure 5, the operator can transfer the workspace in on mode through switching the mode of the hand rest function. Regardless of the mode, the orientation and gripping sections interact with the operator using impedance control.

## 3 EXPERIMENTS AND RESULTS

### 3.1 Evaluation of the positioning accuracy of the hand rest function in the direction of gravity

**FIGURE 6:**
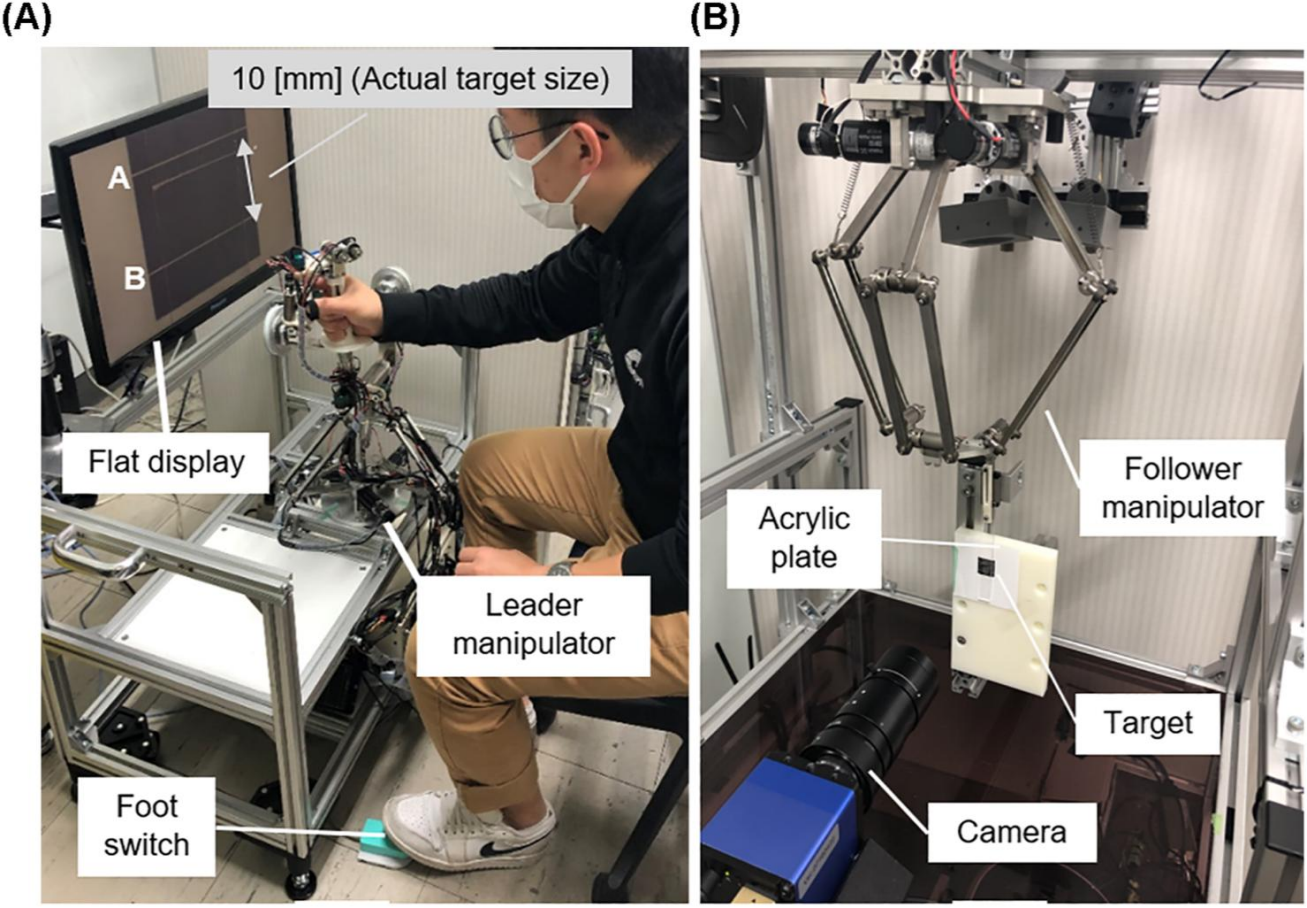
Overview of the evaluation of positioning accuracy: (A) leader part; (B) follower part

One of the main purposes of using a hand rest is to support the hand motion at the intended position in the direction of gravity. We evaluated the positioning accuracy of the hand rest function in the direction of gravity using teleoperation, as shown in Figure 6.

The follower manipulator, which has 3 DoFs with a delta mechanism, was operated to move in one direction according to only the positional information in the gravity direction of the translation section of the leader manipulator. An acrylic plate attached to the follower manipulator was connected to the experimental target with a miniature linear guide; thus, the movement of the follower manipulator can be observed in one direction. One line was drawn on the acrylic plate, and two lines (A and B) were drawn on the target at 10‐mm intervals with the same thickness. Operators were able to manipulate the translation section of the leader manipulator while pressing the foot switch (off mode, $Z_{\text{off}}:M=0.77\;\left[\text{kg}\right],\;\;B=0.001\;\left[\text{Ns}/\text{mm}\right]$) and fix it in that position by releasing the foot (on mode, $Z_{\text{on}}\;=\;Z_{\text{off}}\;\times \;10^{5}$). The expanded two‐dimensional vision of the target was provided to the operator through the flat display. The robotic systems were controlled using Ubuntu 18.04 with a control period of 1 ms, and communicated using local area network. The time delay of the teleoperation was less than 3 ms.

**FIGURE 7:**
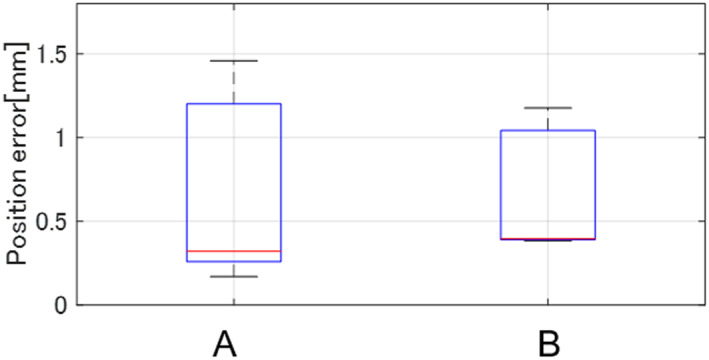
Results of the evaluation experiment (maximum error)

Five male participants (age: 24 ± 2) who operate the system for the first time were asked to manipulate the leader manipulator, as well as align and stop the line on the follower manipulator to the lines on the target three times. The result of each participant was obtained as the maximum error in three trials where the hand rest function turned on in each line on the experimental target. Figure 7 shows the experimental results of all participants on two lines of the experimental targets. During the experiment, all participants made less than 1.5 mm of error in every trial. These results show that the position of the hand rest could be arranged at the intended position in the direction of gravity using the hand rest function.

### 3.2 Pointing experiment

In the previous evaluation, we confirmed that the position of the hand rest corresponding to the pivot point of the leader manipulator could be arranged at the intended position in the direction of gravity using the hand rest function. However, in operating the hand rest function, the conditions on a plane perpendicular to the direction of gravity should be considered as the hand supported by the hand rest is not completely restrained. In this section, we evaluated the operating condition of the leader manipulator considering the conditions of the hand rest function during precise work. General surgeries are performed by placing surgical tool tips to the correct position (3 DoFs in translation) with the intended posture (3 DoFs in orientation). Especially, the highest possible precision in position and posture is required in microsurgical procedures. On the basis of these requirements, we evaluated the effectiveness of manipulating the leader manipulator via pointing experiments with teleoperation under multiple finger and hand movements and conducting a survey regarding preferred conditions.

The robotic systems for microsurgery have been developed to deal with submillimetric surgical targets such as vessels with a diameter between 0.3 and 0.8 mm, using scaling down motions.^7^ The pointing experiment was design to organise an environment for submillimetric tasks by performing positioning operations on experimental targets with a diameter about 1 mm without scaling down motions.

**FIGURE 8:**
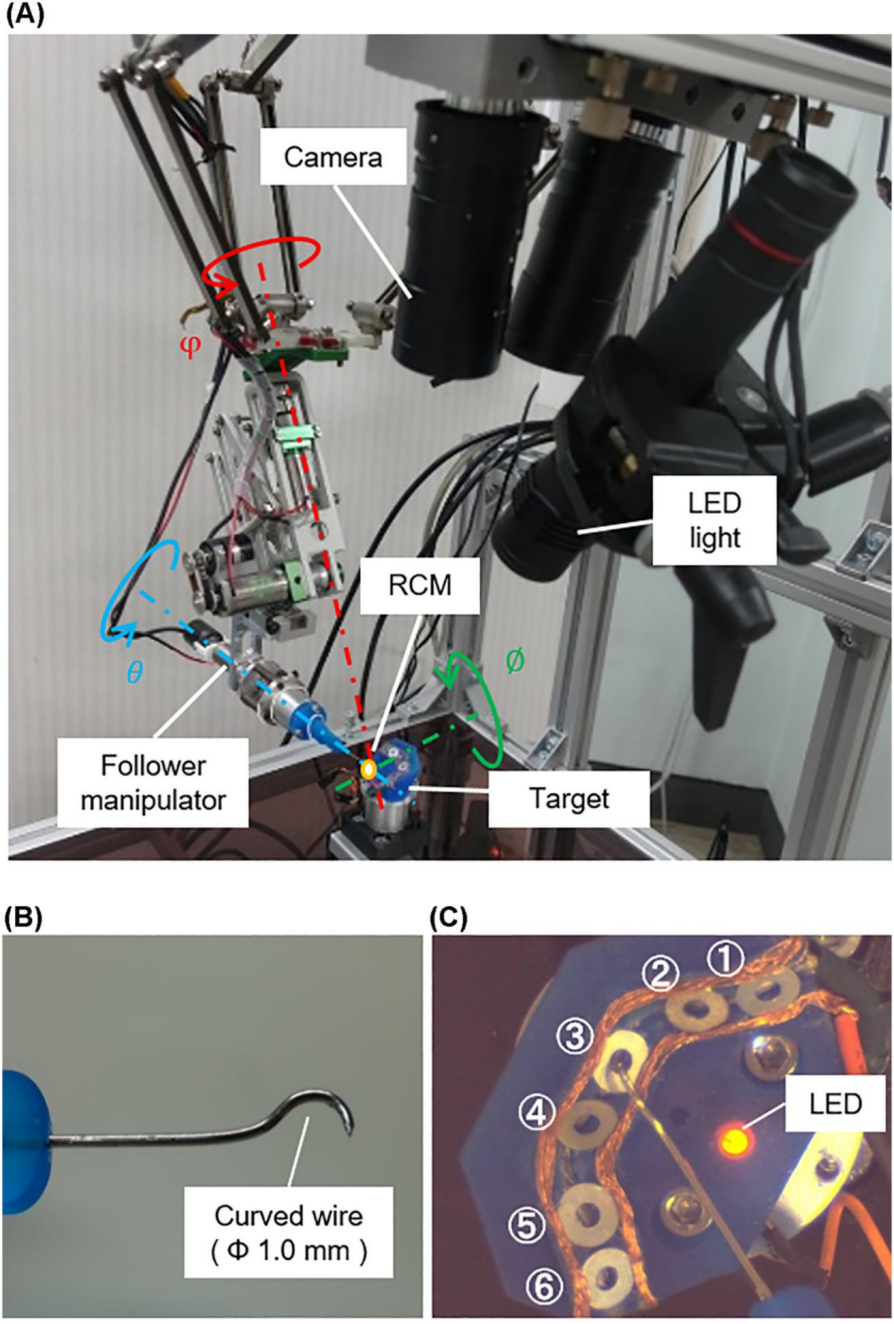
Follower manipulator in the pointing experiment: (A) Overview of the follower part; (B) tip of the follower manipulator and (C) experimental target

**FIGURE 9:**
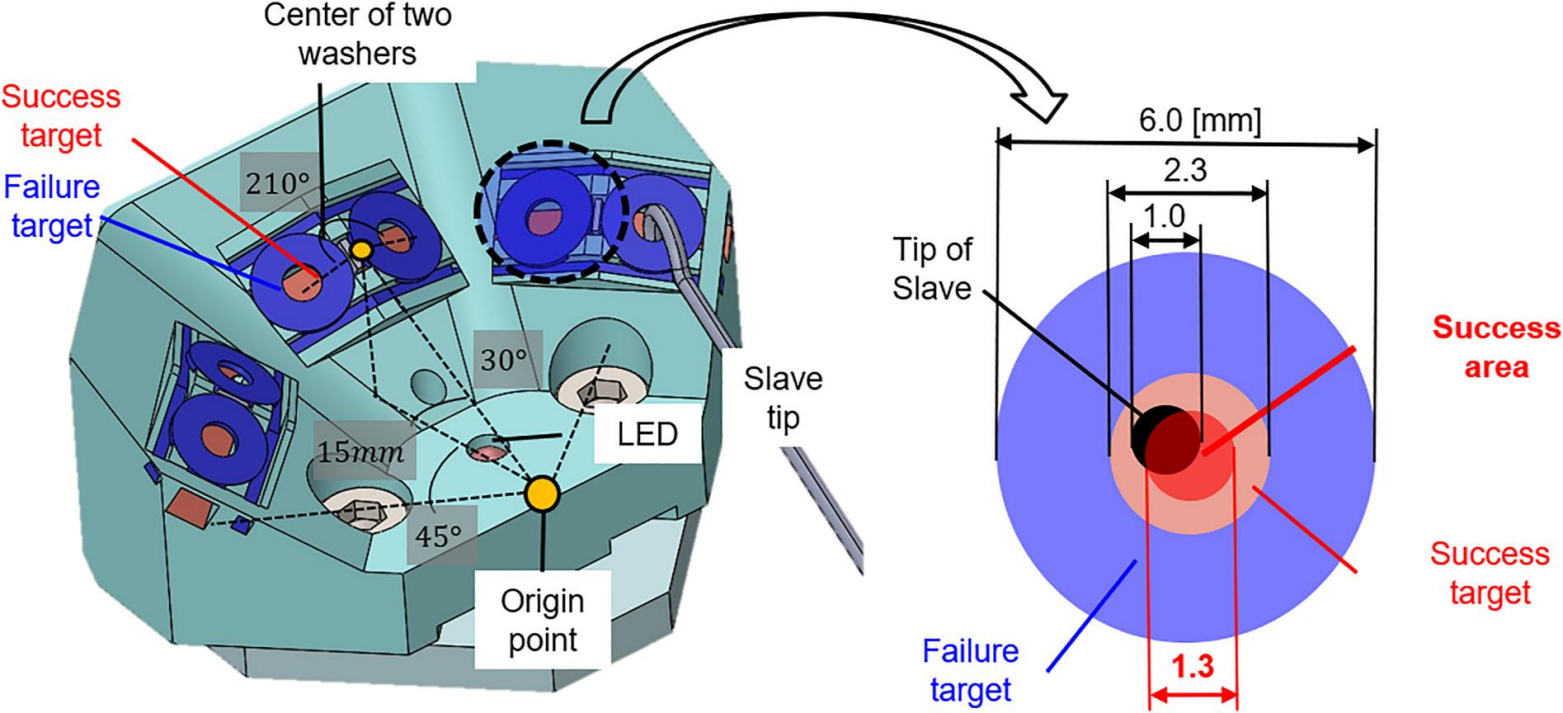
Experimental target of the pointing experiment

Figure 8A shows an overview of the follower manipulator in the pointing experiment adopting the delta mechanism as the translation section (3 DoFs) and the gimbal mechanism that has a remote centre of motion (RCM) as the orientation section (3 DoFs). Curved steel wire with 1.0 mm diameter was mounted on the end effector, as shown in Figure 8B, and the tip of the steel wire was in accordance with the RCM of the follower manipulator. We used harmonic drive gears (Harmonic Drive Systems, INC, CSF‐2XH Series) as reduction gears in each motor of the follower manipulator. The positioning resolution of the follower manipulator, which mainly relies on the translation section, was less than 5 μm. Figures 8C and 9 present the experimental target. The target comprised the failure target, which involves the obstacles to get through to access the success target, and the success target to contact for the experimental task, and the light‐emitting diode (LED). When the tip of the follower manipulator having a potential difference with the target contacted the success target, the LED in the experimental target lighted on. The success area where the success target was pointed without contact with the obstacle existed as a circle with a diameter of 1.3 mm. The six success areas were arranged at different positions and angles. Accordingly, the probability of contacting the obstacles increased when the posture of the follower manipulator was inadequate; hence, it was required to accord to the optimal postures of the follower manipulator for each path to access the success target in the experimental task. Each success area was numbered from ① to ⑥ counterclockwise.

**FIGURE 10:**
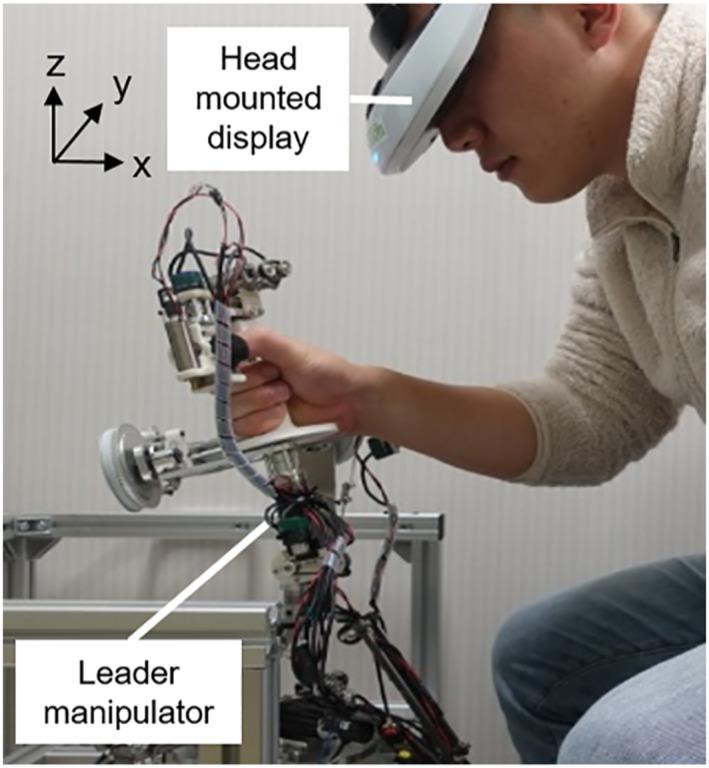
Overview of the leader part in the pointing experiment

Figure 10 shows an overview of the leader part in the pointing experiment. An operator could acquire the three‐dimensional vision obtained from the stereo camera in the follower part with the head mounted display. The operator could adjust the height, position of the seating chair, and the distance between the manipulator and the operator.

The experimental conditions for manipulating the leader manipulator comprised two variables. One was the condition on the finger movement: the case that the finger movement was constrained with the mechanical stopper, which fixed the position and posture of the gripping point from the handle, and the case of using the finger movement by releasing the stopper. The other one was the operating condition on the *x*–*y* plane, where the *z*‐axis in the coordinate system of the leader was the direction of gravity and the hand rest function. The position of the hand rest on the *z*‐axis was fixed together by operating the hand rest function. The conditions in the second variable of the hand rest were classified into three stages according to the applied impedance value in the admittance control, and the condition that the hand rest was fixed with the overwhelmingly large impedance value was added to compose four conditions. An increase in the impedance of the hand rest function indicates an increase in force required, making the movement difficult. The low impedance value (*L*) was set at a value at which the manipulator could be manipulated most lightly and stably (*Z*).

Table 1 shows the experimental conditions of the pointing experiment. Since it was impossible to perform the experimental task in the condition that both the finger and hand rest movements were restricted, the experiment was performed at seven conditions from CL to rest was fixed (RF), excluding the combination of CF.

**TABLE 1:** Experimental conditions for manipulating the leader manipulator

	Low: Z (L)	Medium: Z×10 (M)	High: $\;Z\;\times \;10^{2}$ (H)	Fixed: $Z\;\times \;10^{5}$ (F)
Constrained (C)	CL	CM	CH	CF
Released (R)	RL	RM	RH	RF

*Note*: Z:M=0.77[kg],B=0.001[Ns/mm].

Abbreviations: CH, constrained finger movement with the high impedance value; CL, constrained finger movement with the low impedance value; CM, constrained finger movement with the medium impedance value; RF, released finger movement with the fixed hand rest; RH, released finger movement with a high impedance value hand rest function; RL, released finger movement with the low impedance value hand rest function; RM, released finger movement with the medium impedance value hand rest function.

The follower manipulator was operated in accordance with the positional and postural information from the leader. The ratio of the motion in the follower to that in the leader was 1:1. Therefore, in the teleoperation, the follower manipulator was controlled by following the equations below:

(7) $${\dot{X}}_{f}\;=\;{\dot{X}}_{l}$$

(8) $$P_{f}\;=\;P_{l}$$

where ${\dot{X}}_{l}$ and ${\dot{X}}_{f}$ are the velocities of the leader and follower at Cartesian coordinate and $P_{l}$ and $P_{f}$ are the postures of the leader and follower.

The experiment was conducted according to the standard ethical practices for human experiments and approved by the ethics committee of the Tokyo Institute of Technology. The robotic systems of the leader and follower were operated as in the previous experiment. Fifteen participants (12 males and 3 females, age: 26 ± 4), who operate this system for the first time, were instructed to manipulate the leader manipulator and point the success target with the tip of the follower manipulator by avoiding the failure target. The order of pointing target was from ① to ⑥ and the reverse order consecutively. The participants proceeded to the next step after confirming the contact with the success target through the light of the LED. The experimental conditions were randomly applied to each participant and all participants practiced the experimental tasks beforehand to reduce the possibility of the influence of the learning curve.

**FIGURE 11:**
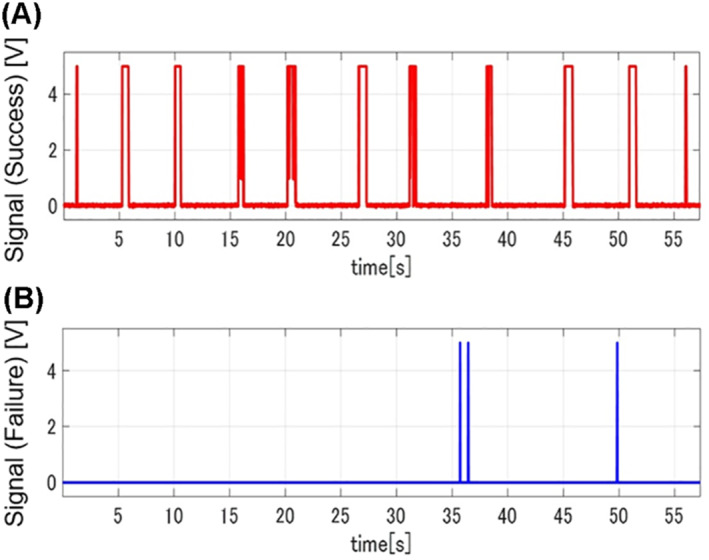
Contacting information between the tip of the follower manipulator and the targets: (A) contacts with the success target and (B) contacts with the failure target (one participant, RH)

**FIGURE 12:**
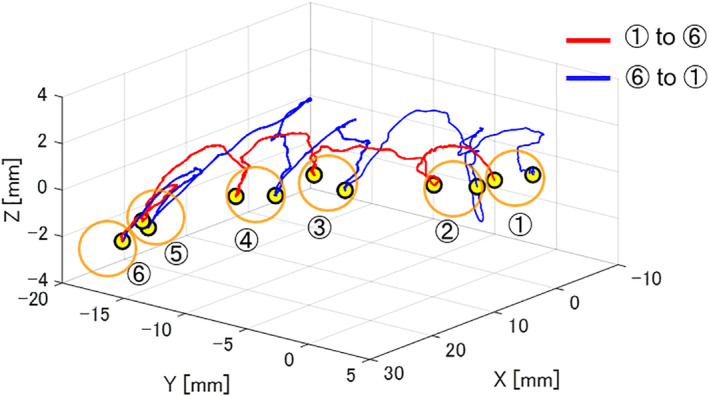
Positional information of the input motion in the leader (one participant, RH)

We evaluated the effectiveness of the manipulating method in terms of time required to complete the tasks, failure number, length of the trajectory of input motion, ratio of the wrist/finger movement to the overall input motion in the leader, and the preference score. As shown in Figure 11, contact between the tip of the follower manipulator and the target was detected as electrical signals. The time required was obtained from the time duration between the time from pointing the starting point to pointing the finishing point based on the signals between the follower tip and the success target. The number of failures was counted as the number of contacts between the follower tip and the failure target during the operation. The length of the trajectory was calculated from the positional information of the input motion to the leader. Figure 12 shows the example of the trajectory of the input motion in the leader. The input motion comprised the movement of the hand rest and the fingertip movement caused by the wrist/finger motion. The movement of the hand rest could be obtained by tracking the movement of the translation section of the leader manipulator. The trajectory of the fingertips caused by the wrist/finger motion could be calculated from the positional and postural information of the orientation and gripping sections. The participants were instructed to give a score from 1 to 7 according to the preference rank of the manipulation conditions after completing the experiments; one point was given to the least preferred condition, whereas seven points were given to the most preferred condition.

### 3.3 Results of pointing experiment

**FIGURE 13:**
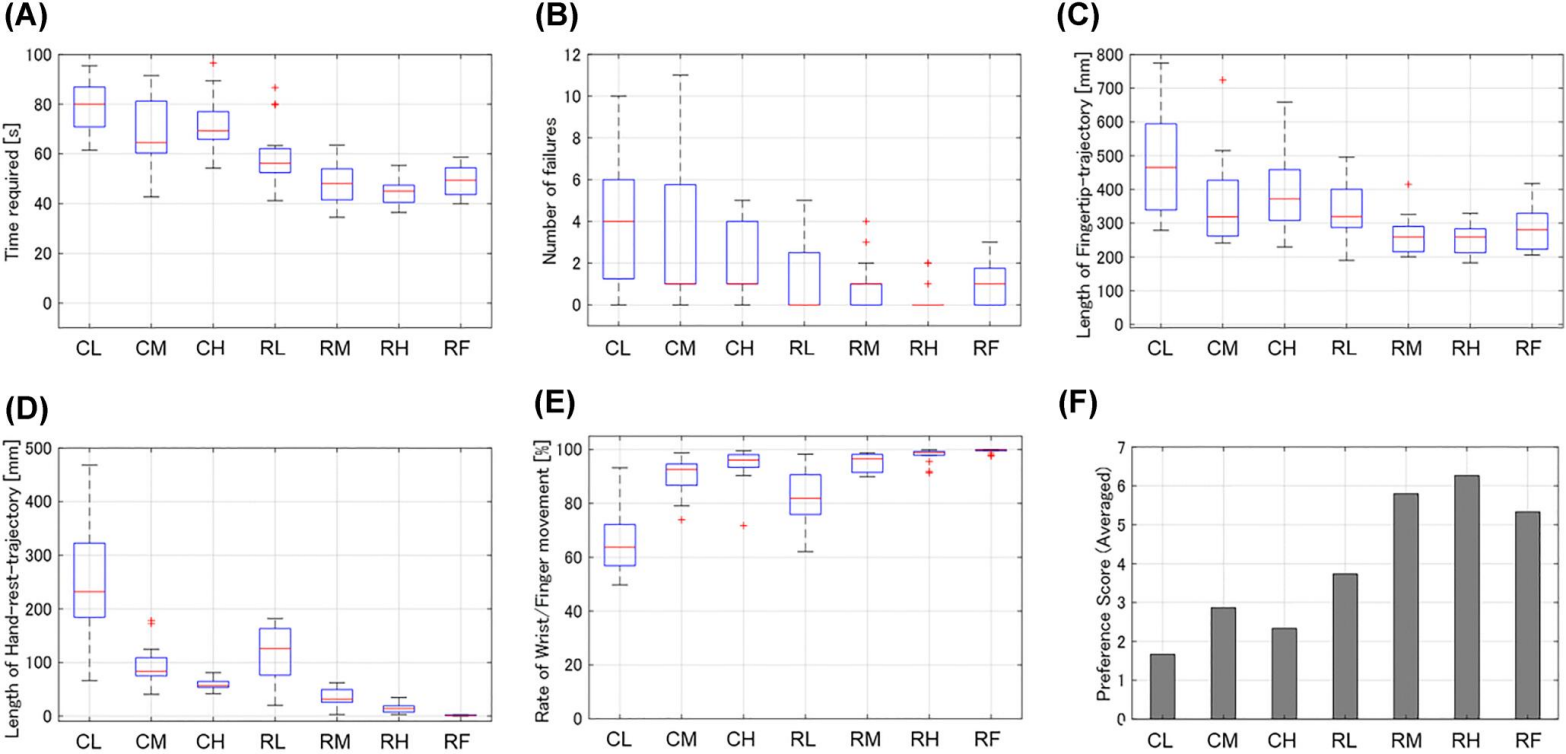
Results of the pointing experiments: (A) time required, (B) number of failures, (C) length of the trajectory of input motion (D) length of the trajectory of hand rest, (E) ratio of the wrist/finger movement, (F) preference scores of the conditions in the pointing experiments

Figures 13A–C, respectively, present the experimental results of the time required, number of failures and length of the trajectory of the input motion in the leader. Tables 2, 3, 4 present the results of the significance test on them according to the condition of the hand rest function in each condition of the finger movement, respectively.

**TABLE 2:** P‐values of the significance tests for the required time

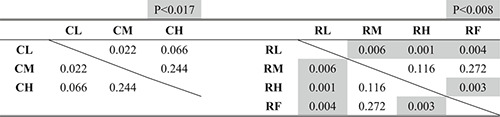

**TABLE 3:** P‐values of the significance tests for the number of failures

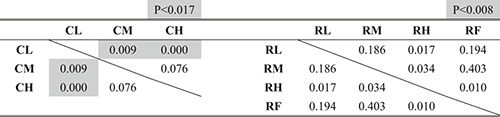

**TABLE 4:** P‐values of the significance tests for the trajectory of the input motion

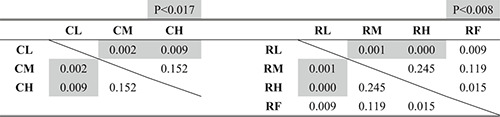

To determine the statistical differences of the hypothesis that the condition, which recorded less (shorter) averaged results, recorded less (shorter) overall results, we considered the Bonferroni corrected *p*‐value from the *p*‐value of less than 0.05 (one‐tailed) as significant.^22^ We found that the case of using the finger movement (R) required shorter time, fewer failures, and shorter trajectory than the case where the finger movement was constrained (C), regardless of the condition of the hand rest function (p<0.05).

Figure 13D,E show the experimental results of the length of the trajectory of the hand rest and the ratio of the wrist/finger movement to the total input motion, respectively. Table 5 presents the results of the significance test on the ratio according to the condition of the hand rest in each condition of the finger movement. The case of using the finger movement (R) required a higher rate of the wrist/finger movement to the total input motion than in the case that the finger movement was constrained (C) under the same condition of the hand rest function (p<0.05).

**TABLE 5:** P‐values of the significance tests for the ratio of the wrist/finger movement to the total input motion

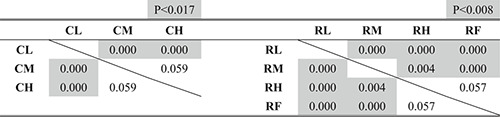

Figure 13F shows the averaged preference scores of the manipulating conditions. Based on the results, the upper and lower groups of the preference scores can be separated, depending on whether the finger movement was possible. In all conditions, using the finger movement (R) scored higher than any condition that the finger movement was constrained (C), regardless of the condition of the hand rest function.

The analysis of the results according to the operating condition of the hand rest function is presented in the subsequent texts.

- Time required

As shown in Figure 13A, the time required decreased with increased applied impedance in the case of using the finger movement (R), whereas there was no clear trend in the case of constrained finger movement (C). Based on the results in Table 2, using the finger movement with the fixed hand rest (RF) required the same amount of time with the condition with the medium value (RM) with no statistically significant differences and longer time than that applied the high impedance value (RH) with statically significant differences. Consequently, the condition of constraining the finger movement with a low impedance value (CL) recorded the longest time required, whereas that of using the finger movement with a high impedance value (RH) recorded the shortest time required.

- Number of failures

As shown in Figure 13B, the number of failures decreased with an increase in the applied impedance to the hand rest function in both cases with and without finger movements (C and R). Based on the results in Table 3, we found no statistically significant differences between the conditions using the finger movement. Consequently, the condition of constraining the finger movement with the low impedance value (CL) recorded the most, and the all conditions of using the finger movement recorded the least number of failures.

- Length of the trajectory of the input motion

As shown in Figure 13C, the trajectory of the input motion in both cases with and without finger movements (C and R) shortened when the higher impedance value (M or H) was applied, compared with the condition with a low value (L). However, based on the results in Table 4, we found no statistically significant differences between the results of the medium (M) and high value (H) cases. In the case of using finger movement, there are no statically significant differences between the result of the condition with the fixed hand rest (RF) and other conditions (RL, RM and RH), whereas there are clear differences between the condition with a low value (RL) and the conditions with a higher values (RM and RH). Consequently, constraining the finger movement with the low impedance value (CL) recorded the longest, whereas the cases using the finger movement except with the condition applied with the low impedance value (RM and RH) recorded the shortest trajectory of the input motion in the leader.

- Ratio of the wrist/finger movement to the overall input motion

As shown in Figure 13D,E, the rate of the wrist/finger movement to the total input motion became higher with an increase in the applied impedance to the hand rest function, whereas the trajectory of the hand rest shortened with and without finger movements (C and R). Based on the results in Table 5, we found no statistically significant differences between the conditions that applied the medium (CM) and high impedance values (CH) in the case of constraining the finger movement. Moreover, there were no statistically significant differences between the condition applying the high impedance value (RH) and the condition where the hand rest was fixed (RF). Consequently, constraining the finger movement with the low impedance value (CL) recorded the lowest ratio of the wrist/finger movement to the total input motion, whereas using the finger movement with the high impedance value (RH) and the fixed hand rest (RF) recorded the highest ratio.

- Preference

As shown in Figure 13F, the preference scores given by the participants increased with the impedance of the hand rest function with the finger movement (R), whereas the preference scores without the finger movement increased in the order of the medium (CM), high (CH), and low impedance values (CL). The score for using the finger movement with the fixed hand rest (RF) was between those with the medium (RM) and low impedance value (RL) of the hand rest function.

## 4 DISCUSSION

### 4.1 Hand rest function in leader manipulator

In this study, we proposed a leader manipulator by integrating the motion input and hand rest functions and evaluated the operating conditions of the hand rest function. The hand rest function integrated into the leader manipulator is advantageous for not only spatial convenience but also repositioning the hand rest compared with using conventional hand rests through external devices. By manipulating one device instead of two separate devices without interference or misalignment between the devices, an operator can interact with the leader manipulator proactively through a simple operation, such as a foot switch. Effectively repositioning the hand rest could contribute to both the surgical procedure and the setup procedure of the microsurgery. Fine manipulation with the hand rest and large working range from the repositioning of the hand rest could improve the quality of surgery. Additionally, improving the surgery quality in a certain scale factor can lower the optimal scale factor and reduce useless motion in the leader. Simply repositioning the hand rest can reduce the trial and error in the initial procedure, particularly if the working range of the surgical robot is narrow.

We experimentally verified the optimal condition in the on mode of the hand rest function in this study. In our future work, we will focus on the operating system, such as a stability on switching the state of the hand rest function to verify its effectiveness on extending over a wide working range. In addition, we will quantitatively evaluate the operators' fatigue of the leader manipulator in order to clarify its effectiveness. Ultimately, we will comprehensively compare the operating performance of the existing and the proposed leader manipulators for microsurgical procedures.

### 4.2 Optimal operating condition of hand rest function

We verified that the position of the hand rest in the direction of gravity could be arranged at the intended position using the hand rest function of the leader manipulator. Moreover, we evaluated the operating conditions of the hand rest function on the plane perpendicular to the direction of gravity by carrying the pointing experiment. The condition on the finger movement in the pointing experiment reflected the aspects of the conventional leader manipulators applying a power or a pinch grip. As an example, the case that the finger movement was constrained is reminiscent of a haptic device adopting a power grip such as a sigma.7 (Force Dimension, Inc.) that determines the input motion initiated by the wrist and entire arm without finger movement.^23^

In the case that the finger movement was constrained, the conditions of the higher impedance values of the hand rest function showed better performance than those with the low impedance at which the leader manipulator could be manipulated most lightly and stably. Between the results of the conditions of the medium and high impedance values, there were no statistically significant differences. The highest preference score was given to the condition with the medium impedance value. Thus, the optimal impedance value lies between the medium and high values and is close to the medium value in this study.

In the case of using the finger movement, the overall performance with the higher ratio of wrist/finger movement to the total input motion became better with an increase in the applied impedance to the hand rest function. Between the results for the medium and high impedance values, there were no statistically significant differences. The condition with the fixed hand rest and that with the medium impedance value showed the same performance, which was worse than that with the high impedance value. The preference scores decreased in the order of the conditions with high impedance, medium impedance, fixed hand rest, and low impedance. Therefore, we can assume that the movement of the pivot point with the proper impedance is required for dexterous work on a plane perpendicular to the direction of gravity, and the optimal impedance value for the movement lies between the medium and high values in this study.

Based on the overall results of the pointing experiment, the differences between the results in the case of constraining the finger movement and that in the case of using the finger movement through the hand rest function was clear in all aspects of the time required, number of failures, trajectory, and preference. In the use of hand rest, finger movement is an essential element for precise work. In the case that the finger movement is constrained, it would be better to support the arm instead of the hand for operation since using a hand rest may reduce intuitive operation. Consequently, the optimal manipulating condition of the proposed leader manipulator was the condition with hand rest function and high impedance, which did not fix the hand rest completely. Similar to finding an optimal impedance for admittance control, the optimal impedance value depends on the manipulator, and it is necessary to select an adequate impedance for manipulating the leader manipulator and operating the hand rest function under the rough direction introduced in the study.

## 5 CONCLUSIONS

The advantages in fine manipulation with the optimised hand rest function and large working range from the repositioning of the hand rest have the potential to improve the quality of robotic microsurgery. The main findings of this study are summarised as follows:

- We proposed a leader manipulator, which integrated the motion input and hand rest functions and adopted a combined‐grip scheme. The hand rest function, which generates the stable pivot point, was realised by switching the impedance value in admittance control with a simple operation of the interface, such as a foot switch, in the translation section of the leader manipulator.
- With the teleoperation, we verified that the position of the hand rest in the direction of gravity could be arranged at the intended position in the evaluation experiment. Additionally, we conducted a pointing experiment for evaluating the manipulating method during precise positioning by focussing on the conditions of the hand rest function on a plane perpendicular to the direction of gravity under several conditions in terms of time required, number of failures, length of trajectory, and preference.
- We verified that the optimal manipulating condition of the leader manipulator was the condition that the hand rest function adopted sufficiently high impedance and did not fix the hand rest completely and the operator could use the finger movement on the basis of the hand rest.

## CONFLICT OF INTEREST

The authors do not have conflicts of interests in relation to this manuscript.

## Data Availability

Data openly available in a public repository that issues datasets with DOIs.
